# Spatial variations and mechanisms for the stability of water use efficiency in China

**DOI:** 10.3389/fpls.2023.1254395

**Published:** 2023-09-21

**Authors:** Xiaojuan Xu, Jing Liu, Fusheng Jiao, Kun Zhang, Yue Yang, Jie Qiu, Yingying Zhu, Naifeng Lin, Changxin Zou

**Affiliations:** ^1^ Nanjing Institute of Environmental Sciences, MEE, Nanjing, China; ^2^ School of Geography, Nanjing Normal University, Nanjing, China

**Keywords:** ecological restoration, water use efficiency, climate change, stability, driving factors

## Abstract

A clearer understanding of the stability of water use efficiency (WUE) and its driving factors contributes to improving water use efficiency and strengthening water resource management. However, the stability of WUE is unclear. Based on the EEMD method, this study analyses the spatial variations and mechanisms for the stability of WUE in China, especially in the National Forest Protection Project (NFPP) areas. It is found that the stable WUE was dominated by non-significant trends and increasing trends in China, accounting for 33.59% and 34.19%, respectively. The non-significant trend of stable WUE was mainly located in the Three-North shelterbelt program area, and the increasing trend of stable WUE was in Huaihe and Taihu, Taihang Mountains, and Pearl River shelterbelt program areas. Precipitation and soil moisture promoted the stable WUE in these project areas. The unstable WUE was dominated by positive reversals or negative reversals of WUE trends. The positive reversals of unstable WUE were mainly located in the Yellow River shelterbelt program areas, which was promoted by temperature and radiation, while the negative reversals of unstable WUE were mainly distributed in the Yangtze River and Liaohe shelterbelt program areas, which were mainly induced by saturation water vapor pressure difference (VPD). Our results highlight that some ecological restoration programs need to be improved to cope with the negative climate impact on the stability of WUE.

## 1 Introduction

Water use efficiency (WUE) is an objective evaluation index of water-carbon coupling for an ecosystem, which is defined as the ratio of carbon sequences (i.e., gross primary production (GPP)) to water loss (i.e., evapotranspiration (ET)) (Beer et al., 2009; Jiang et al., 2019; Ma J. et al., 2019; Liu et al., 2020). WUE is not only an important index of the coupled terrestrial carbon-water cycle, but also one of the important parameters reflecting the impact of global changes on terrestrial ecosystems (Hatfield and Dold, 2019; Yang L. et al., 2022). Uncovering the changes in the stability of the ecosystem WUE can provide important technical and policy implications for water conservation and carbon budgets (Cao et al., 2020). A large interannual variable in water use efficiency can lead to the instability of ecosystem functions and pose a serious challenge to nature-based climate solutions (Xue et al., 2015; Zhang et al., 2016; Wang et al., 2022). Thus, spatial variations and mechanisms for water use efficiency stability have become a foregrounded and topical issue for ecosystems.

The interannual variables of WUE are stable in the absence of climate and human drivers of change (Rödenbeck et al., 2018; Piao et al., 2020). However, the driving factors and their interactive effects influence GPP and ET in different ways, making the interannual variables in WUE threaten the stability of WUE (Ma J. et al., 2019). The instability in WUE considerably challenges the sustainability of the carbon-water cycle (Yu et al., 2021). The essence of WUE instability is that WUE has weak resistance to climate fluctuation (Wang et al., 2022). Both climate and ecosystems have obvious spatial differences, thus, WUE trends are nonlinear and their stability has a significant spatial variation (Piao et al., 2020). In areas with severe climate fluctuations or where WUE is sensitive to climate fluctuations, the stability of WUE is usually low (Liu et al., 2017; Ma J. et al., 2019). It provides a rare opportunity to explore the response of ecosystem functioning to climate change (Piao et al., 2020). However, there still exists uncertainty on the WUE stability response to climate change (Belmecheri et al., 2021). Thus, reducing the uncertainty is critical for an accurate future carbon-water cycle and its response to climate change (Belmecheri et al., 2021; Migliavacca et al., 2021).

Previous studies have shown that WUE change is influenced by climate change, such as CO_2_, solar radiation, temperature, precipitation, saturated water vapor pressure, and soil moisture (Liu et al., 2020; Gonsamo et al., 2021; Wang M. et al., 2021). Precipitation is one of the principal indirect driving factors affecting WUE variables (Zhang et al., 2020). Precipitation changes directly affect the transpiration and evaporation of the ecosystem, and indirectly affect the carbon uptake process of plants by regulating the soil water content (Piao et al., 2020; Zhang et al., 2020). In addition, drought-induced reduction in vegetation production and WUE was offset by driving factors, such as warming climate and ecological restoration projects (Huang J. et al., 2015; Zhang et al., 2016; Ma J. et al., 2019). Numerous studies have shown that vapor pressure difference (VPD) is a key factor and has been demonstrated to have negative influences on the WUE (Beer et al., 2009; Ma J. et al., 2019). It is revealed that the increase in CO_2_ concentration will increase the photosynthetic rate, while the transpiration rate will be weakened or have no significant effect, which will lead to the increase of vegetation WUE, with a significant CO_2_ fertilization effect (El Masri et al., 2019; Ma J. et al., 2019; Gonsamo et al., 2021). Both photosynthesis and transpiration are affected by temperature, and show an opposite trend with increasing temperature (Huang J. et al., 2015). When the temperature was low, the photosynthetic rate increased with the increase of temperature, and then gradually weakened after it reached the maximum (Hatfield and Dold, 2019). The main reason was that the enzyme activity was significantly affected when the optimum temperature was reached (Hatfield and Dold, 2019). On the contrary, the increase in temperature will cause an increase in VPD and then increase the transpiration rate of vegetation (Cao et al., 2020). The radiation is one of the important factors of plant photosynthesis, and it also impacts plant WUE (Jiang et al., 2019; Wang et al., 2020). Nevertheless, the driving mechanisms underlying the stability of WUE remain unclear.

The National Forest Protection Project (NFPP) in China is one of the world’s largest ecological restoration projects (Tong et al., 2017; Lu et al., 2018; Huang et al., 2019). It is a major initiative by the Chinese government to mitigate some of the environmental damage caused by rapid economic development through the implementation of a rigorous and creative policy of large-scale conservation (Cai et al., 2020; Ding et al., 2021; Xu et al., 2022a). Currently, some researchers believe that the implementation of the NFPP has led to an increase in vegetation cover in some areas, thus improving the carbon and water cycle and the ecological environment (Tong et al., 2018; Huang et al., 2019; Yang Y. et al., 2022). Another group of scholars believe that the implementation of the NFPP, especially afforestation in some areas, has led to a reduction in soil moisture due to strong forest transpiration and the increased precipitation cannot compensate for the consumption of evapotranspiration, which places a burden on local water resources (Yang et al., 2013; Hai et al., 2022). Therefore, there is an urgent need to reveal the actual trend and stability of WUE and its driving factors in NFPP areas and to provide scientific advice for the implementation of ecological projects.

In this study, we investigated the spatial variations and mechanisms for the stability of water use efficiency in China. Specifically, we aimed to propose three key issues: (1) What are the nonlinear trends of WUE in China, especially in NFPP areas? (2) What is the stability of WUE in China over recent years? (3) Which driving factors are important in determining the stability of WUE in China?

## 2 Materials and methods

### 2.1 Data sources

The terrestrial gross primary production (GPP) data was downloaded from the Global Land Surface Satellite (GLASS) program (http://www.resdc.cn/) (Jiao et al., 2022). It is generated using the Bayesian algorithm ensemble of eight widely-used light-use efficiency models and has been widely used in global carbon cycle assessment (Xu et al., 2022a). The dataset is a global composite product that spans from 1982 to 2015 with a spatial resolution of 0.05°.

The terrestrial evapotranspiration (ET) data was downloaded by the National Science & Technology Infrastructure (http://www.nesdc.org.cn/), with a 0.1° spatial resolution from 1981–2015. To obtain more accurate data, ET data is simulated by a nonlinear complementary Relational model and verified with 13 vorticity covariance measurements and 10 river basin Nash Sutcliffe efficiency measurements, with a range of 0.72-0.94 (Ma N. et al., 2019).

The WUE was calculated as follows:


(1) WUE=GPP/ET

In this study, six driving factors were used to reveal the driving mechanism of the stability of WUE. The soil moisture (SM) and downward shortwave radiation (RAD) were downloaded by the Climatology Lab from 1981 to 2015 with a spatial resolution of 1/24°. Temperature and precipitation were downloaded from WorldCom (https://www.worldclim.org/) from 1981 to 2015 with a spatial resolution of 1 km. Vapor pressure deficit (VPD) was provided by Xu et al. (2021) (http://dx.doi.org/10.1016/j.scitotenv.2022.155086) from 1981 to 2015 with a spatial resolution of 0.1°. CO_2_ was downloaded from the Emissions Database for Global Atmospheric Research (EDGAR) (https://edgar.jrc.ec.europa.eu/) from 1981 to 2015 with a spatial resolution of 0.1°.

### 2.2 Nonlinear method

The linear trends of NEP show an increasing trend with a constant rate of increase. However, the rate of NEP decomposed by EEMD increases with time. Therefore, the EEMD method can reveal the nonlinear trends in WUE (Pan et al., 2018; Xu et al., 2022b). Ensemble Empirical Mode Decomposition (EEMD) is an extension of the Empirical Mode Decomposition (EMD) method (Wu et al., 2007). The decomposition process of EMD is as follows:

First, the discrete extreme points of *X(t)* are interpolated to the entire period with a cubic spline function to obtain the maximum and minimum envelopes, and the arithmetic mean of the upper and lower envelopes (*g*_1_(*t*)) is calculated.


(2) $$g_{1}(t)=X(t)-m_{1}(t)$$

Since *g_1_(t)* is not stable, continue to repeat the above steps:


(3) $$g_{11}(t)=g_{1}(t)-m_{11}(t)$$

If the standard deviation (SD) is less than a given value (usually 0.2), the above iterative process is terminated:


(4) $$SD=\sum_{t=1}^{N}\frac{|g_{1k}(t)-g_{1(k-1)}(t){|}^{2}}{g_{1(k-1)}^{2}(t)}$$

In this way, we extracted the first Intrinsic Mode Function IMF (IMF) component (*c_1_=g_1k_(t)*) from the original data:


(5) $$g_{1k}(t)=g_{1\left(k-1\right)}(t)-m_{1k}(t)$$

*k* is the number of iterations and the rest of the original data is:


(6) $$r_{1}=X(t)-c_{1}$$

Since *r_1_* still contains fluctuations of a longer period, the above iterative process is still repeated, and the *r_i_* is as follows:


(7) $$r_{i}=r_{i-1}-c_{i},i=2,3\cdots n$$

Which is


(8) $$X(t)=\sum_{i=1}^{n}c_{i}+r_{n}$$

*c_i_* is the *i*-th IMF component, and *r_n_* is the residual.

Due to the phenomenon of frequency mixing in the EMD method, the EEMD method was developed (Wu and Huang, 2009). The EEMD method introduces white noise with a certain signal-to-noise ratio into the original time series for EMD decomposition, and the IMFs obtained by each decomposition are then aggregated. The EEMD method not only greatly improves the modal aliasing defect of EMD, but also avoids the instantaneous noise that the original data may carry.

Repeat equations 2-8, with different Gaussian white noise series assed to *X(t)*, Finally, the original signal is decomposed into a series of *IMF_i_(c_i_)* components with frequencies from high to low and a residual *r_n_*.

The EEMD trends in WUE as a specific time t is defined as the value increase in *r_n_* since the start time, that is trend(t)=*r_n_* (*t*)-*r_n_* (*1981*). The trends as their changing rates can be calculated (Pan et al., 2018):


(9) $$Rate_{trend}(t)=Trend(t)-Trend(t-1)$$

The number of Gaussian white noises was set to 100 times, and the amplitude of these noises was set to 0.2 standard deviations of the raw data after considering the take-off between the decomposition robustness and the required computing time.

**Figure 1:**
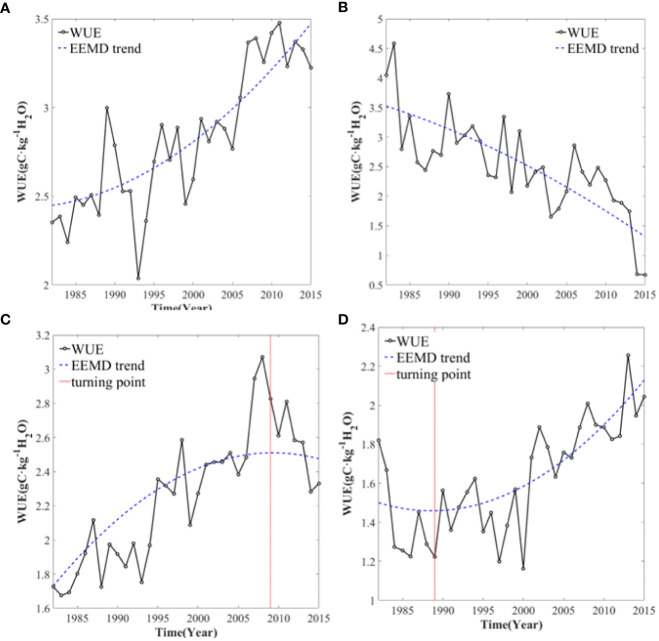
The nonlinear trends of WUE. **(A)** increasing trends; **(B)** decreasing trends; **(C)** negative reversals; **(D)** positive reversals.

To test whether the trend is significant, the EEMD decomposition of Gaussian white noise is verified based on the Monte Carlo method (Pan et al., 2018). The non-significant trend of WUE is assumed to show no trend over time. The significantly non-linear trend of the WUE is divided into the following four categories: increasing trends (**Figure 1A**), decreasing trends (**Figure 1B**), negative reversals (**Figure 1C**), and positive reversals (**Figure 1D**).

### 2.3 Stability method

Based on nonlinear trends detected by EEMD, excluding the trends with insignificant changes, we computed the interannual anomalies of WUE by removing their nonlinear trends. The standard deviation of interannual anomalies of WUE was identified as the stability of WUE (Wang et al., 2022). A higher standard deviation suggested lower stability. Additionally, the stability of WUE was classified into 6 classes (nonsignificant stable, stable, relatively stable, generally stable, relatively unstable, and unstable) based on natural breaks. The natural interval method is based on natural groupings inherited from the data. When creating classification intervals, similar values are grouped most appropriately and differences between classes are maximized. Elements are divided into classes, for which their boundaries are set at locations where the differences in data values are relatively large.

### 2.4 Attribution analysis method

Multiple regression analysis was used to extract the impacts of the main drivers on the stability of WUE (Sun et al., 2015; Liu et al., 2018; Chu et al., 2019). We select 6 driving factors (CO_2_, temperature (TEM), precipitation (PRE), soil moisture (SOIL), radiation (RAD), and vapor pressure deficit (VPD)) as the explanatory variables (Cai et al., 2015; Sun et al., 2015). Based on min-max normalization, all the driving factors were standardized in advance. The multiple regression analysis method is as follows:


(10) $$\begin{array}{c} SWUE_{pre}=aSWUE^{CO2}+bSWUE^{TEM}+cSWUE^{PRE}\\ +dSWUE^{RAD}+eSWUE^{SOIL}+fSWUE^{VPD}+g \end{array}$$

Where *SWUE_pre_* indicates the predicted stability of WUE, *SWUE^CO^* ^2^, *SWUE^TEM^*, *SWUE^PRE^*, *SWUE^RAD^*, *SWUE^SOIL^*, *SWUE^VPD^* represent SWUE variations that are driven by CO_2_, TEM, PRE, RAD, SOIL, VPD, which were also standard deviations of the interannual anomalies without long-term nonlinear trends (Wang et al., 2022). *a*–*f* are the regression coefficients. The absolute value of the regression coefficient can represent the relative importance of the driving factors, and *g* is the regression constant. In this study, the largest regression coefficient of the multiple regression is the main driving factor on the stability of *SWUE*.

## 3 Result

### 3.1 The WUE trend in China and eight ecological restoration areas

**Figure 2:**
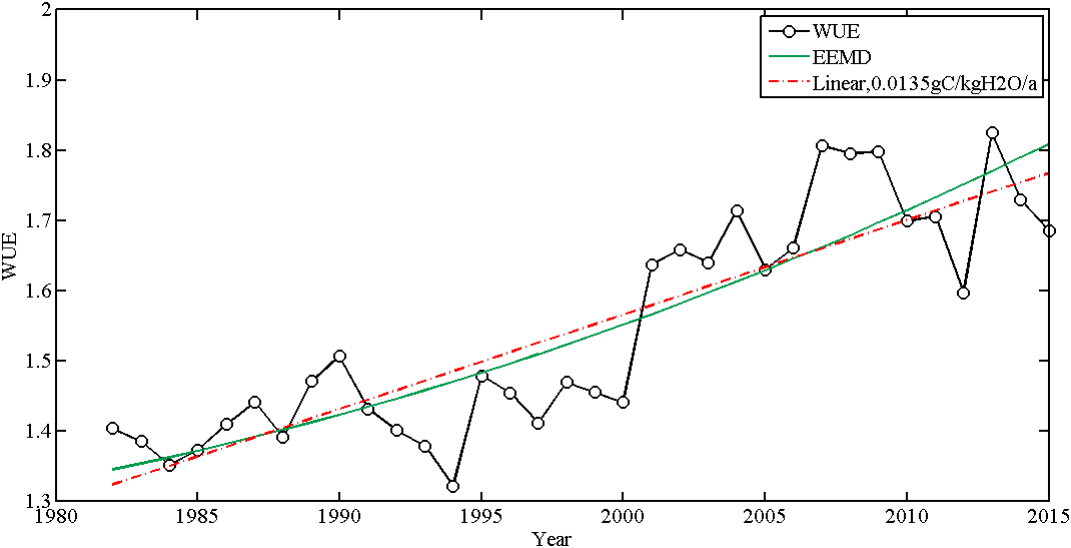
The linear and nonlinear trend of WUE in the whole of China based on linear regression method and EEMD method.

**Figure 3:**
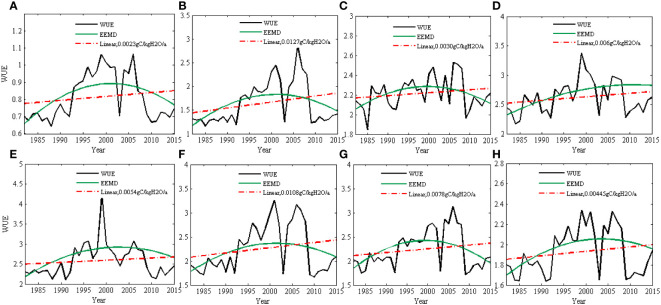
The linear and nonlinear trend of WUE in eight NFPP areas based on linear regression method and EEMD method (**A**: Three-north areas; **B**: Yellow River areas; **C**: Yangtze River shelterbelt program areas; **D**: Pearl River areas; **E**: Liaohe River areas; **F**: Taihang Mountains areas; **G**: Huaihe and Taihu areas; **H**: coastal areas).

Based on the linear method (**Figure 2**), WUE had an increasing trend from 1982 to 2015 with an average rate of 0.0135gC/kgH_2_O/yr. WUE also had a nonlinear trend detected by EEMD and the increased rate was beyond the linear rate after 2005. In the National Forest Protection Project (NFPP) area (**Figure 3**), the WUE had increasing trends, the growth rate of WUE was the fastest in the Yellow River (at a rate of 0.0127 gC/kgH_2_O/yr) and Taihang Mountains (at a rate of 0.0108 gC/kgH_2_O/yr) shelterbelt program areas. It was the slowest in the Three-North shelterbelt program areas (at a rate of 0.0023 gC/kgH_2_O/yr) and the Yangtze River shelterbelt program areas (at a rate of 0.0030 gC/kgH_2_O/yr). There is a tendency for WUE to increase, with the rate of increase peaking around 2000-2005 and decreasing thereafter. It indicated that although ecological restoration projects has significantly improved water use efficiency, there is a risk of WUE reduction in the future.

**Figure 4:**
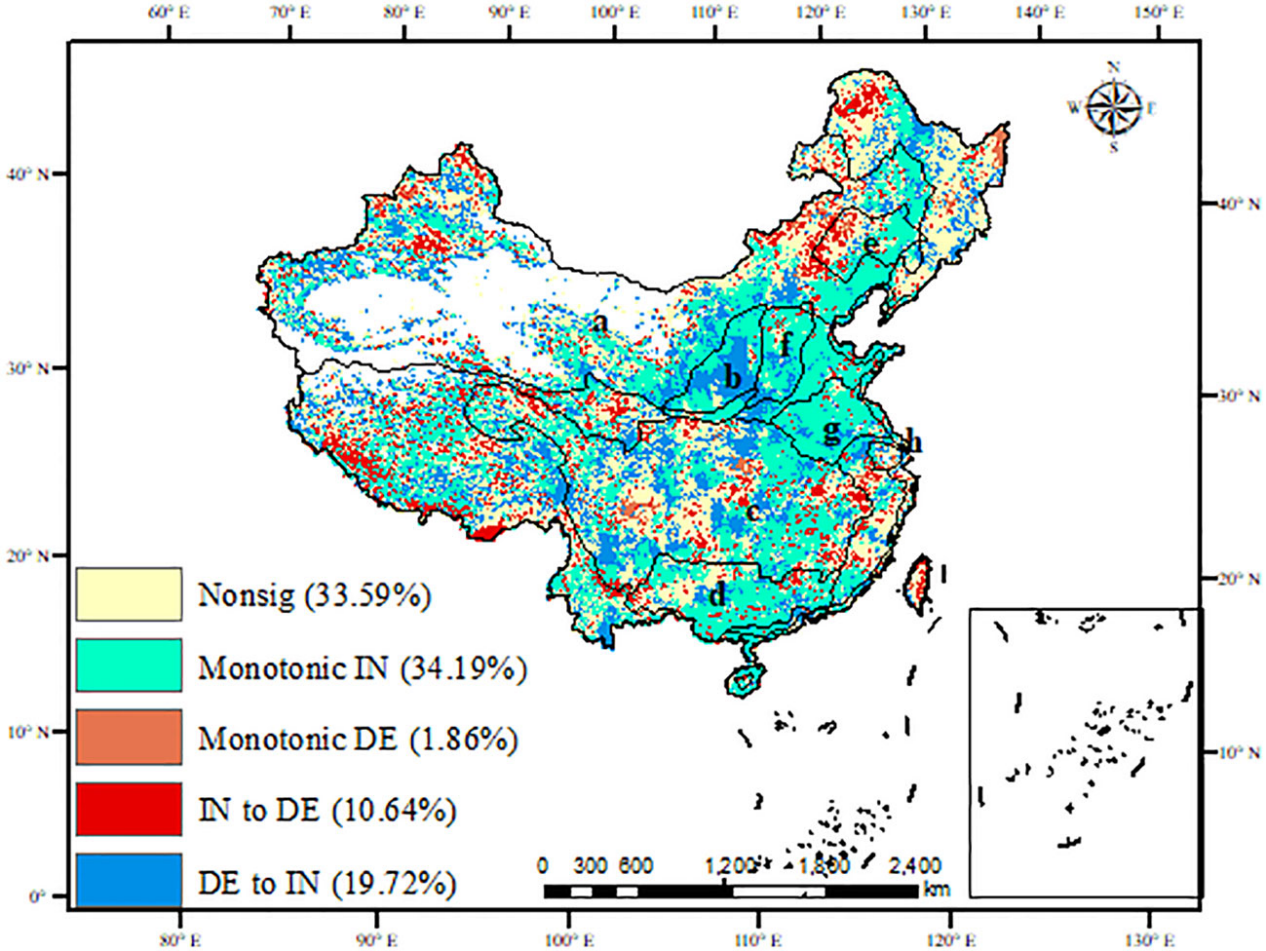
The spatial distribution of the nonlinear trend of WUE in China (Monotonical IN: increasing trends; IN to DE: decreasing trends; IN to DE: negative reversals; DE to IN: positive reversals; A: Three-north areas; B: Yellow River areas; C: Yangtze River shelterbelt program areas; D: Pearl River areas; E: Liaohe River areas; F: Taihang Mountains areas; G: Huaihe and Taihu areas; H: coastal areas).

In China (**Figure 4**), 33.59% of WUE was nonsignificant, which is assumed to be no trend over time and was mainly located in the Northwest, Northeast, and Tibetan Plateau. 34.19% and 19.72% of WUE had increasing trends and positive reversals, which are mainly distributed in the North China Plain and the Pearl River Basin (**Table 1**). The decreasing trends and negative reversals of WUE only accounted for 1.86% and 10.64%, respectively, located in the Yangtze River basin. In eight NFPP areas, WUE was dominated by increasing trends in the Pearl River, Taihang Mountains, Huaihe, and Taihu shelterbelt program areas, accounting for 57.90%, 69.42%, and 72.25%, respectively. It had positive reversals in the Yellow River shelterbelt program areas, accounting for 44.63%. However, in the Yangtze River, the WUE was dominated by negative reversals, which accounted for 51.35%.

**Table 1:** The percentage of nonlinear trends of WUE in China and eight NFPP areas.

Region	Nonsig	Monotonical IN	Monotonical DE	IN to DE	DE to IN
Whole China	33.59	34.19	1.86	10.64	19.72
Three-North	35.10	29.75	2.63	10.28	22.24
Yellow River	6.89	47.45	0	1.02	44.63
Yangtze River	18.54	17.94	0.89	51.35	11.28
Pearl River	23.92	57.90	0.05	8.03	10.10
Liaohe River	35.96	38.94	2.92	14.53	7.62
Taihang Mountains	8.20	69.42	0.06	3.65	18.68
Huaihe and Taihu	10.33	72.25	0.96	3.36	13.10
Coastal	23.12	51.50	0.73	9.92	14.72

* Nonsig, nonsignificant trends; Monotonical IN, increasing trends; IN to DE, decreasing trends; IN to DE, negative reversals; DE to IN, positive reversals.

### 3.2 The spatial distribution of the WUE stability

**Figure 5:**
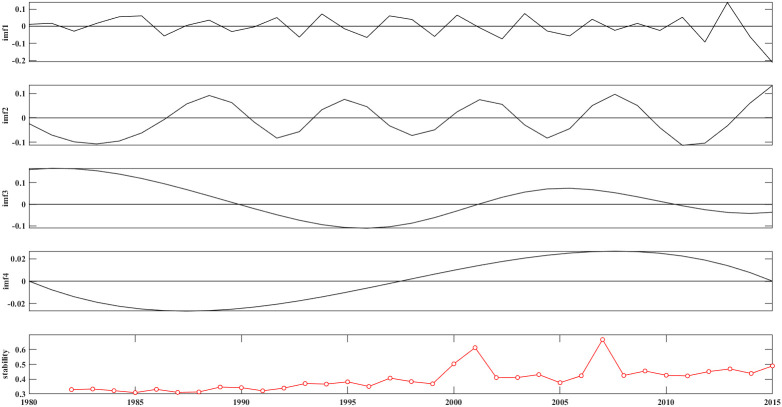
EEMD decomposition for averaged WUE in China (interannual anomalies (IMF1-IMF4); residual trend; stability).

EEMD decomposes WUE into four interannual variations and a residual (**Figure 5**), with periods of 2.7, 6.5, 27, and 38-year time scales. The SWUE (standard deviation of interannual variation) ranged from 0.30 to 0.67. The SWUE were relatively stable (ranging from 0.30-0.40) with small interannual fluctuations before 2000. There was a large instability of WUE after 2000. To be more specific, it increased sharply in 2000, 2001, and 2007, with the greatest volatility in 2007.

**Figure 6:**
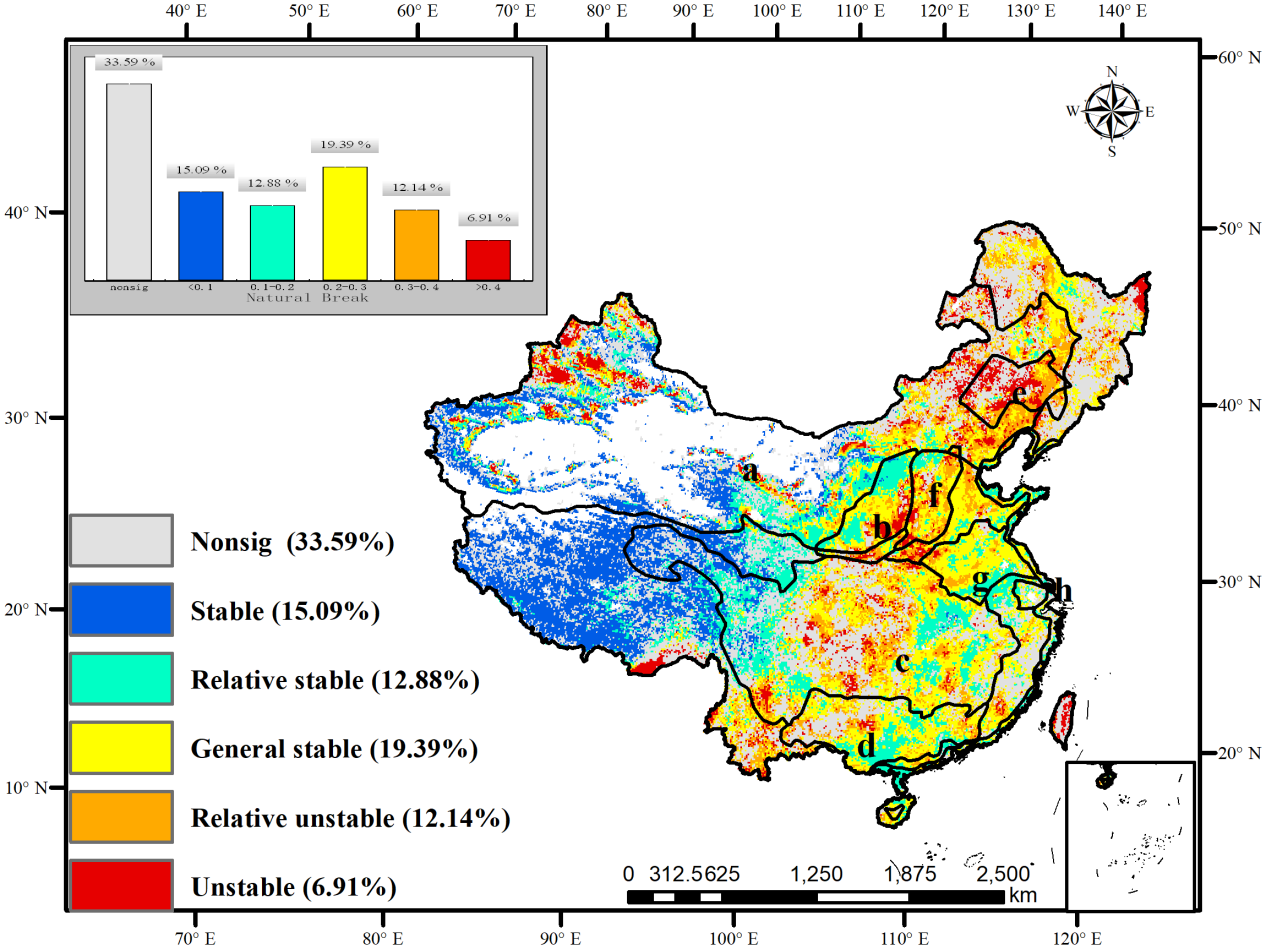
The spatial distribution of stability in WUE in China (non sig: non-significant stability of WUE. A: Three-north areas; B: Yellow River areas; C: Yangtze River shelterbelt program areas; D: Pearl River areas; E: Liaohe River areas; F: Taihang Mountains areas; G: Huaihe and Taihu areas; H: coastal areas).

The six classes of SWUE, namely non-significant stable, stable, relatively stable, generally stable, relatively unstable, and unstable in China, account for 33.59%, 15.09%, 12.88%, 19.39%, 12.14%, and 6.91%, respectively (**Figure 6** and **Table 2**). Overall, WUE was stable in the northwest and Tibetan Plateau, where the trend was mainly nonsignificant; while it was unstable in the northeast, southwest, and Yangtze River basin, where the WUE trends were dominated by negative reversals. In NFPP areas, the WUE was stable in Three-North shelterbelt program areas and the stable and relatively stable WUE accounted for 17.57% and 12.03%, respectively. the relatively stable WUE accounted for 32.81% in Coastal shelterbelt program areas. However, in the Liaohe and Taihang Mountains shelterbelt programs areas, the percentage of the relatively unstable and unstable WUE accounted for 57.00% and 45.62%, respectively. In Taihang Mountains shelterbelt program areas, the WUE was dominated by monotonically increasing trends and positive reversals, while the WUE usually experienced negative reversals in Liaohe River areas. It is indicated that WUE with nonlinear trends (positive reversals and negative reversals) are generally unstable, which may be influenced by climatic or anthropogenic factors, leading to positive shifts or negative feedback.

**Table 2:** The percentage of stability of WUE in China and eight NFPP areas.

Region	Nonsig	Stable	Relative stable	General stable	Relative unstable	Unstable
Whole China	33.59	15.09	12.88	19.39	12.14	6.91
Three-North	35.41	17.57	12.03	13.56	12.00	9.70
Yellow River	6.89	0.67	28.89	34.24	17.55	11.77
Yangtze River	34.47	7.89	16.62	23.48	13.16	4.38
Pearl River	23.95	0.03	19.10	41.21	13.44	2.27
Liaohe River	35.93	0.00	0.39	6.68	25.98	31.02
Taihang Mountains	8.25	0.00	10.65	35.47	32.79	12.83
Huaihe and Taihu	10.38	0.00	22.8	55.46	10.42	1.26
Coastal	22.74	2.54	32.81	34.47	6.45	0.98

### 3.3 The dominant climatic factors on the SWUE

**Figure 7:**
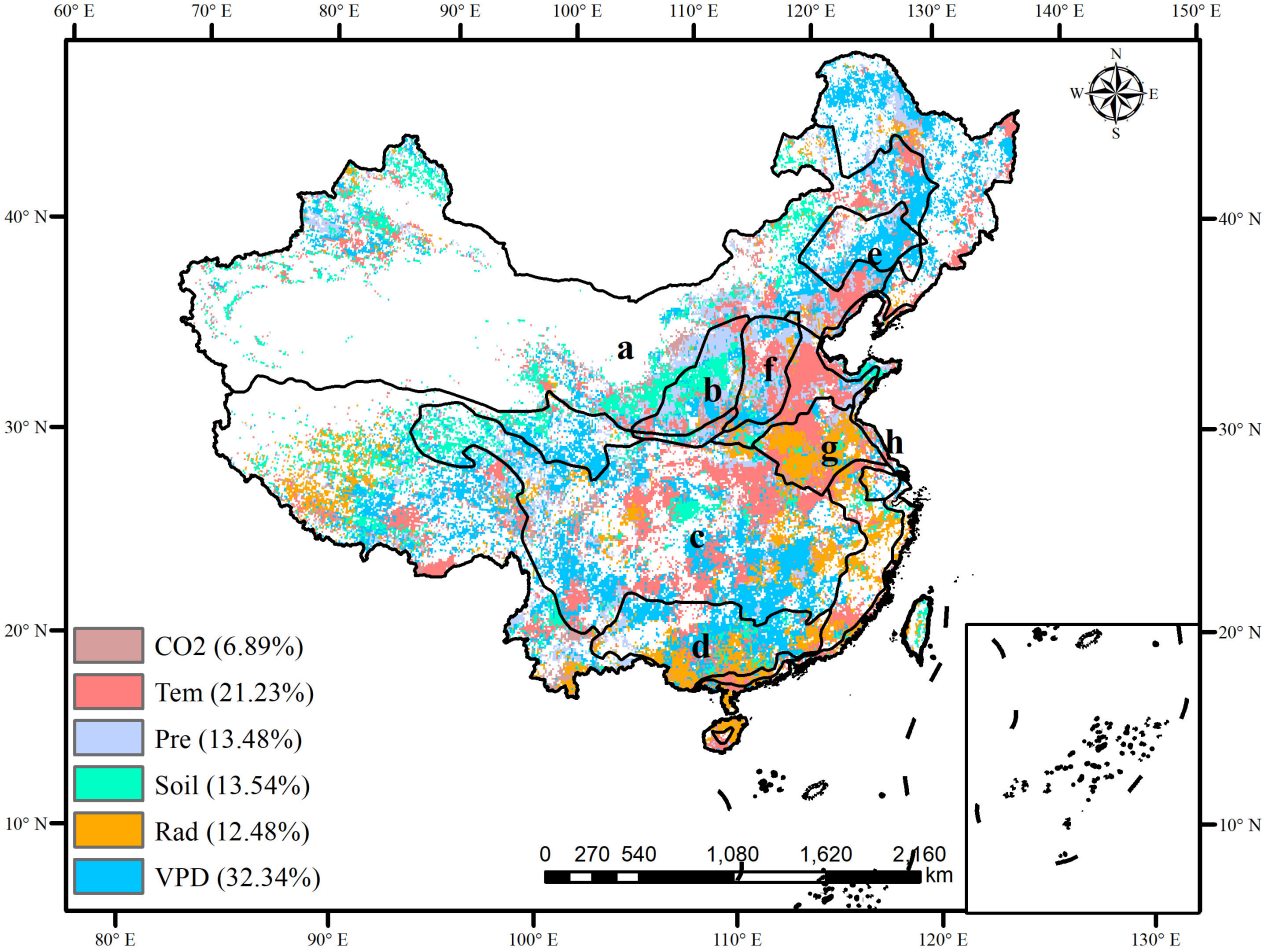
The dominant driving factors of WUE in China (A: Three-north areas; B: Yellow River areas; C: Yangtze River shelterbelt program areas; D: Pearl River areas; E: Liaohe River areas; F: Taihang Mountains areas; G: Huaihe and Taihu areas; H: coastal areas).

To investigate the mechanism of climate fluctuations on SWUE in different regions of China, we analyzed the spatial distribution of the main climatic factors on the SWUE in China (**Figure 7**). The results show that the spatial variation of SWUE was mainly influenced by VPD in southwestern and northeastern China and along the Yangtze River, where the WUE was unstable. Temperature and solar radiation were the dominant factors for interannual fluctuations of WUE in the North China Plain and central Yangtze River, where the WUE was relatively unstable. In the NFPP areas, VPD was the main driving factor for the interannual variables of WUE in the Yangtze River, Pearl River, and Liaohe River shelterbelt program areas, accounting for 38.25%, 49.38%, and 65.85%, respectively (**Table 3**). This indicates that the WUE was affected by atmospheric drought in these ecological project areas, which led to low SWUE. Precipitation and soil moisture were the main driving factors for the interannual variables of WUE in the Yellow River and Tibetan Plateau, where the WUE was stable. Temperature and radiation promoted interannual variables of WUE in the Taihang Mountains and Yangtze River shelterbelt program areas, where the WUE was relatively unstable. The overall contribution of CO_2_ to SWUE was low in eight NFPP areas. It indicated that VPD, temperature, and radiation lead to unstable changes in WUE, while precipitation and soil moisture lead to stable changes. Therefore, differences in the dominant climate factors in different regions need to be considered when exploring the influence of climate on the SWUE in China.

**Table 3:** The percentage of driving factors of WUE in China and eight NFPP areas.

Region	CO_2_	Tem	Pre	Soil	Rad	VPD
Whole China	6.89	21.23	13.48	13.54	12.48	32.34
Three-North	7.18	16.54	16.29	28.17	4.27	27.55
Yellow River	8.48	12.54	32.56	25.83	1.64	18.95
Yangtze River	4.18	27.21	8.89	9.82	11.65	38.25
Pearl River	5.95	13.88	5.54	5.25	25.54	49.38
Liaohe River	5.26	12.56	12.54	1.53	2.25	65.86
Taihang Mountains	3.92	32.86	33.79	4.89	6.26	18.28
Huaihe and Taihu	8.15	26.75	4.89	4.15	43.87	12.19
Coastal	3.59	35.57	11.53	8.89	25.29	15.16

* Tem, temperature; Pre, precipitation; Soi, soil moisture; Rad, radiation; VPD, vapor pressure deficit.

## 4 Discussion

### 4.1 The nonlinear trend of WUE

The core meaning of WUE can be summarized as the ratio of productivity to water consumption (El Masri et al., 2019; Jiao et al., 2022). A deep understanding of the WUE trend is an important entry point for coupling the water-carbon cycle, energy conversion, resource use, and climate change issues, especially in the ecological restoration areas (Jiao et al., 2022). Basing on linear method, previous studies revealed that the WUE shows an increasing trend with a rate of 0.0025 gC kg/H_2_O/yr globally (Xue et al., 2015; Yang L. et al., 2022; Zhao et al., 2022). In this study, the WUE was also dominated by increasing trends (34.19%) in China, while decreasing trends only accounted for 1.86%. Additionally, the nonlinear trends of WUE were detected by the EEMD method. 19.72% of WUE had positive reversals while 10.64% exhibited negative reversals, which is rarely explored by the linear methods (Jiao et al., 2022). Ignoring non-linear changes in WUE may lead to an overestimation of ecosystem productivity and an underestimation of water deficit (Hatfield and Dold, 2019). Thus, revealing the nonlinear trends of WUE provided a deep understanding of ecosystem functioning (Jiao et al., 2022). In eight NFPP areas, WUE was dominated by increasing trends or positive reversals. The time of turning points mainly occurred in 2000-2005, aligning well with the implementation of the NFPP (Chen et al., 2019). The restoration project has increased the area of forest and scrub at high WUE levels, and significantly reduced the area of farmland and grassland at low WUE levels, thus increased the overall WUE. This shows that the implementation of ecological restoration projects will improve the sustainability of WUE to a certain extent (Ding et al., 2021). However, in the Yangtze River shelterbelt program areas, the negative reversals of WUE accounted for 51.35%, indicating that the Yangtze River basin is facing reduced productively and drought risks, which limits crop production and triggers grassland fires secondary disasters, such as grassland fires and crop pests and diseases (Venkatappa et al., 2021).

### 4.2 The stability of WUE

Although the spatial distribution of the WUE trend has been determined using linear or nonlinear trends in previous studies, the stability of WUE remained unclear (Rödenbeck et al., 2018; Jiao et al., 2022). Jiao et al. (2022) obtained the WUE stability through the contribution of inter-annual variables to the secular trend, and revealed that the WUE was strongly stable in the north and Loess Plateau while interannual variables were found in the southwest. The trend itself can reflect the stability of the WUE. In this study, based on the EEMD method, the standard deviation of residual trends of WUE is used as the indicator of stability, and the spatial variations of SWUE were compared and analyzed. The change in WUE experienced large inter-annual fluctuations after 2000, especially from 2000 to 2005. Overall, compared to northwestern China and the Tibetan Plateau, the WUE in eastern China was much more unstable. A previous study showed that most of the ecological functioning has been improved in China (Jin et al., 2018). In this study, the unstable WUE experienced positive reversals or negative reversals. The traditional linear methods that ignore non-linear trends exaggerate the stability of WUE (Hao et al., 2021). Thus, there are hidden risks beneath apparent ecosystem degradation, and ecosystem improvement or degradation may be limited and exaggerated (Pan et al., 2018).

Previous studies have revealed that ecological restoration promotes the improvement of ecosystem functioning (Yang et al., 2017; Song et al., 2022). In this study, the WUE was stable with increasing trends in the Pearl River, Huaihe and Taihu, coastal shelterbelt program areas. Although WUE was unstable in the Yellow River and Taihang Mountains shelterbelt program areas, it experienced positive shifts. Previous studies have shown that the ecological restoration program contributed to a significant increase in vegetation productivity in the Yellow River and Taihang Mountains shelterbelt program areas (Xue et al., 2022). It is indicated that most ecological restoration projects in China enhance carbon sequestration thus leading to increasing trends or positive shifts in WUE (Xue et al., 2022). However, some ecological restoration may inhibit or reverse the trend in WUE (Lu et al., 2018). Our result demonstrated that WUE was unstable and may be easily altered from increasing to decreasing in the Yangtze River shelterbelt program areas. In the Yangtze River shelterbelt program area, the massive planting of trees stimulates an increase in the water demand of the forest, which in turn absorbs large amounts of soil water and stimulates increased evapotranspiration (Zhang et al., 2016). The simultaneous increase in productivity and evapotranspiration can lead to fluctuating changes in WUE, especially during the implementation phase of the ecological project from 2000-2005 (Tong et al., 2019). In addition, the Yangtze River is in the East Asian monsoon climate zone and is significantly influenced by the monsoon, with frequent drought disasters (Hong et al., 2014). Studies have shown that drought events in the Yangtze River have begun to increase and intensify in recent years, which deeply affected vegetation productivity (Horion et al., 2016). In areas with significant human influence and stable WUE, such as the southeastern coast, there is little room for further improvement of WUE stability. Therefore, achieving the sustainability of WUE may depend on reducing anthropogenic carbon emissions in these areas (Wang et al., 2022). Therefore, the spatial heterogeneity in WUE stability underscores the importance of implementing management strategies according to the local condition.

### 4.3 Driving mechanism of WUE

Climate change has a profound impact on the functioning of ecosystems and, as a result, on the coupled cycles of carbon and water (Liu et al., 2020). Some climatic factors influence the trend and stability of WUE, such as CO_2_ concentration, VPD, temperature, precipitation, soil moisture, etc. (Cheng et al., 2017; Hatfield and Dold, 2019). In our study, the stability variation of WUE is mainly caused by precipitation and soil moisture, while instability is mainly induced by saturation water vapor pressure difference, temperature, and solar radiation in China.

Previous studies suggested that the atmospheric water demand, represented by VPD overrode other climatic factors exerting dominantly negative effects on WUE change in alpine meadow ecosystems (Cheng et al., 2017). In this study, the VPD played a dominant role in controlling the instability of the WUE trend in the Yangtze River and Liaohe River shelterbelt program areas, where the WUE experienced negative reversals. By controlling plant stomatal activity, atmospheric drought can affect carbon acquisition and water transpiration losses (Konings et al., 2017). In recent years, China has undergone an increase in atmospheric vapor pressure deficit (Lopez et al., 2021). VPD plays a dominant role in controlling ET in these areas, with higher VPD leading to a dramatic increase in ET (Liu et al., 2020). Additionally, high VPD should lead to partial stomatal closure and suppression of photosynthetic rates (Ding et al., 2018). All these effects could lead to a negative response of WUE to changes in VPD (Cao et al., 2020). This negative impact is becoming stronger in terms of the severity and extent of the effects, indicating that atmospheric drought is becoming increasingly harmful to productivity (Ding et al., 2018). Therefore, in areas where VPD has led to a decline in WUE, the impact of atmospheric drought on vegetation should be closely monitored and the negative impact of atmospheric drought on vegetation should be reduced through artificial measures, such as artificial rainfall and irrigation.

Temperature and radiation are the dominant controlling factors on the instability of WUE in the Taihu and Huaihe and coastal shelterbelt program areas. In the Taihu Lake and Huaihe River shelterbelt program area, the increase of temperature promotes the increase in WUE, while in some coastal areas, the increase of temperature suppresses the increase in WUE and shifts it from decreasing to increasing. The appropriate increase in temperature prolongs the growth period of plants, leading to a higher increase in GPP than ET, resulting in a monotonic increase in WUE in the Huaihe and Taihu shelterbelt program areas. However, in coastal areas, especially in subtropical areas, the temperature increase promotes ET much more than GPP, resulting in negative reversals and large interannual fluctuations in WUE. There is a threshold for the effect of temperature on water utilization and a too high or too low temperature can harm plant WUE (Hatfield and Dold, 2019). When the temperature is below the threshold, WUE increases with increasing temperature, while when the temperature is above the threshold, WUE shows a negative relationship with temperature (Hatfield and Dold, 2019). The main reason is that enzyme activity is significantly affected when the optimum temperature is reached (Huang M. et al., 2015). Conversely, an increase of temperature causes an increase in VPD and thus increases the transpiration rate of vegetation (Xue et al., 2015). Xue et al. (2015) found that globally, WUE tended to increase linearly with temperature in the cooler regions, reaching a maximum at 18.5°C and decreasing thereafter. Therefore, high temperatures can cause instability in WUE with negative reversals. Thus, more attention should be paid to the high temperature, especially heat waves. In addition, with the economically developed eastern coast and the rapid expansion of urbanization, vegetation productivity is dominated by decreasing or increasing to decreasing trends, leading to negative reversals and instability in WUE (Liu et al., 2023).

Precipitation and soil moisture promoted the stability of WUE in the Yellow River shelterbelt program areas. Precipitation and soil moisture in these areas are relatively low and are crucial elements determining ecosystem composition, structure, and function (Zhang et al., 2020). Interannual variation in vegetation productivity in semi-arid regions is closely related to interannual variables of drought and precipitation (Zhang et al., 2020; Yang L. et al., 2022). Moderate rainfall could offset the effect of drought and keep the stability of WUE in the Yellow River program area (Cai et al., 2020; Wang M. et al., 2021). Previous studies revealed that WUE was positively correlated with precipitation and specific humidity (Xue et al., 2015; Wang H. et al., 2021). Liu et al. (2020) suggested that increased soil moisture contributed to a positive trend in WUE in humid and high latitudes of northern China, which could also enhance carbon sequestration because of the increased water availability (Liu et al., 2020). In addition, the Yellow River basin is the most effective area for ecological restoration projects in China, such as Natural Forest protection, afforestation, economic compensation, etc., which enhance carbon accumulation and greatly contribute to the increasing trends and stability in WUE (Kou et al., 2021; Zhang et al., 2022).

## 5 Conclusions

The stable WUE was dominated by nonsignificant trends and increasing trends, accounting for 33.59% and 34.19%, respectively. The nonsignificant trend of stable WUE was mainly located in Three-North shelterbelt program areas, and the increasing trend of stable WUE was in Huaihe and Taihu, Taihang Mountains, and Pearl River shelterbelt program areas. Precipitation and soil moisture promoted stable WUE in these project areas. The unstable WUE was dominated by positive reversals or negative reversals of WUE trends. The positive reversals of unstable WUE were mainly located in the Yellow River shelterbelt program areas, which was promoted by temperature and radiation, while the negative reversals of unstable WUE were mainly distributed in the Yangtze River and Liaohe shelterbelt program areas, which was mainly induced by VPD.

## Data availability statement

The original contributions presented in the study are included in the article/Supplementary Material. Further inquiries can be directed to the corresponding authors.

## Author contributions

XX: Conceptualization, Data curation, Funding acquisition, Investigation, Project administration, Resources, Software, Validation, Visualization, Writing – original draft, Writing – review & editing. JL: Investigation, Methodology, Software, Supervision, Resources, Visualization, Writing – original draft. FJ: Investigation, Methodology, Software, Supervision, Resources, Visualization, Writing – original draft. KZ: Methodology, Supervision, Conceptualization, Investigation, Software, Writing – review & editing. YY: Data curation, Formal Analysis, Methodology, Project administration, Supervision, Validation, Writing – original draft. JQ: Writing – original draft. YZ: Data curation, Methodology, Supervision, Writing – original draft, Writing – review & editing. NL: Funding acquisition, Resources, Visualization, Writing – review & editing. CZ: Conceptualization, Funding acquisition, Resources, Visualization, Writing – review & editing.
